# Digital Hesitancy Among Nurses and Physicians in Anesthesia and Intensive Care: A Cross-Sectional Study from Romania

**DOI:** 10.3390/nursrep16080290

**Published:** 2026-08-19

**Authors:** Corina Vernic, Ovidiu Bedreag, Dorina Cristina Sulițanu, Ion Petre, Liliana Ioana Muntean, Sorin Ursoniu

**Affiliations:** 1 Doctoral School, “Victor Babeș” University of Medicine and Pharmacy Timișoara, Eftimie Murgu Square 2, 300041 Timișoara, Romania; dorina.beck@umft.ro; 2 Medical Informatics and Biostatistics, Department III: Functional Sciences, Faculty of Medicine, “Victor Babeș” University of Medicine and Pharmacy Timișoara, Eftimie Murgu Square 2, 300041 Timișoara, Romania; petre.ion@umft.ro; 3 Research Center in Anesthesia and Intensive Care (CCATITM), Faculty of Medicine, “Victor Babeș” University of Medicine and Pharmacy Timișoara, Eftimie Murgu Square 2, 300041 Timișoara, Romania; bedreag.ovidiu@umft.ro; 4 Discipline of Anaesthesia and Intensive Care, Department X: Surgery II, Faculty of Medicine, “Victor Babeș” University of Medicine and Pharmacy Timișoara, Eftimie Murgu Square 2, 300041 Timișoara, Romania; 5 Discipline of Cellular and Molecular Biology, Department II: Microscopic Morphology, Faculty of Medicine, “Victor Babeș” University of Medicine and Pharmacy Timișoara, Eftimie Murgu Square 2, 300041 Timișoara, Romania; muntean.ioana@umft.ro; 6 Department III: Functional Sciences, Faculty of Medicine, “Victor Babeș” University of Medicine and Pharmacy Timișoara, Eftimie Murgu Square 2, 300041 Timișoara, Romania; sursoniu@umft.ro; 7 Centre for Translational Research and Systems Medicine, Faculty of Medicine, “Victor Babeș” University of Medicine and Pharmacy Timișoara, Eftimie Murgu Square 2, 300041 Timișoara, Romania

**Keywords:** health personnel, attitude to computers, diffusion of innovation, nursing informatics, uncertainty, nursing

## Abstract

**Background and Objectives:** Digitalization studies often emphasize perceived benefits while overlooking professionals who cannot determine whether digital tools are useful. This study quantified digital hesitancy among anesthesia and intensive-care professionals, examined its relationship with professional role and experience, and compared it with reported barriers and technology use. **Methods:** A single-centre cross-sectional survey included 161 professionals (80.1% of 201 eligible departmental staff): 55 nurses and 106 physicians. A four-item Uncertainty Index measured inability to appraise digital benefits in patient safety, clinical decisions, management, and education. Barrier Burden and Resource-Efficiency indices were also calculated. Group differences were assessed using nonparametric tests, and predictors of high uncertainty were examined by multivariable logistic regression. **Results:** Nurses had substantially higher uncertainty than physicians (mean Uncertainty Index 1.60 vs. 0.49, difference 1.11, 95% CI 0.80 to 1.42; *p* < 0.001), particularly regarding educational and clinical benefits. Reported barriers were similar across professional groups, with technical problems being the most common. Any reported prior use of training simulators, irrespective of frequency or recency, was markedly lower among nurses than physicians (1.8% vs. 47.2%; absolute gap 45.4 percentage points, 95% CI 35.2 to 55.5; *p* < 0.001). After adjustment, physician status remained strongly associated with lower odds of high uncertainty, whereas overall seniority was not significant. In an exploratory within-nurse analysis, uncertainty was higher among more experienced nurses, reaching its highest level among those with more than 20 years of practice (Spearman ρ = 0.49, 95% CI 0.24 to 0.68; *p* < 0.001); the seniority bands were small (*n* = 7 to 18), so this gradient is hypothesis-generating. **Conclusions:** Digital hesitancy was concentrated among nurses and was distinct from shared technical barriers; within nurses it was higher at greater seniority. Because patient-safety outcomes and intervention effects were not measured, targeted simulator-based training and role-specific feedback should be regarded as hypotheses for prospective evaluation alongside, rather than established alternatives to, generic infrastructure improvements.

## 1. Introduction

The implementation of digital technologies in critical care is now widely understood to be a sociotechnical process rather than a purely technical one, in which the attitudes and competencies of the workforce determine whether a tool delivers value or is quietly abandoned [1]. Frameworks of technology acceptance have repeatedly shown that perceived usefulness and perceived ease of use, not the objective capabilities of a system, drive sustained use [2,3,4]. Yet most critical-care evaluations measure only the affirmative pole of this construct—whether staff believe a tool helps—while neglecting the diagnostically richer signal of professionals who simply cannot judge.

This non-committal stance, which we term digital hesitancy, is conceptually distinct from rejection. The diffusion-of-innovations literature distinguishes active resisters from a larger pool of late adopters who remain uncertain because they lack the exposure, feedback, or mental model required to evaluate an innovation [5]. In healthcare, organizational and interpretive reviews have emphasized that such uncertainty is a marker of incomplete sociotechnical integration rather than of a deficient tool [6,7]. Measuring who is uncertain, and in which domains, may therefore identify the most tractable targets for intervention. A non-committal response may arise either from difficulty in appraising a tool or from having had too little contact with it to appraise at all.

Digital hesitancy should be distinguished from four adjacent constructs with which it is readily conflated. Digital readiness and digital health competence describe an assessed capability to use technology and are operationalised as skills or self-rated proficiency; hesitancy, by contrast, is an epistemic state about the value of a tool and can coexist with entirely adequate technical skill. Technology acceptance models place their outcome on a bipolar evaluative dimension anchored by perceived usefulness and perceived ease of use [2,3,4]; a respondent who cannot appraise a tool does not occupy the negative pole of that dimension but stands outside it, having formed no evaluation at all. Resistance to change denotes an active, motivated and behaviourally expressed stance against an innovation, whereas hesitancy is passive and non-committal. Low computer self-efficacy concerns confidence in one’s own capacity to operate a system [8,9] rather than judgement about what that system achieves for patients. Within the Non-Adoption, Abandonment, Scale-up, Spread and Sustainability (NASSS) framework, hesitancy maps most directly onto the adopter-system domain, in which the value proposition remains unresolved for a particular professional group even where the technology and organizational domains are settled [1,10]. The related notion of technology-induced inertia captures the resulting stasis: continued nominal use of a system without the interpretive engagement that would allow a professional to judge whether it helps. Positioning digital hesitancy in this way clarifies what is novel about it, and identifies the appraisal capacity of specific user groups, rather than infrastructure provision or attitude, as the object of measurement.

Because the present study operationalises hesitancy through ‘cannot appraise’ responses, one caveat should be stated at the outset. Such a response is compatible with at least three distinguishable states: genuine cognitive uncertainty about whether a tool delivers benefit; insufficient exposure, training or direct use to have formed an opinion; and a pragmatic judgement that the technology is peripheral to one’s own role. These states are not separable in a cross-sectional survey, and the interpretation of the Uncertainty Index is bounded accordingly.

There are strong reasons to expect that hesitancy is unevenly distributed across professional roles. Survey research on electronic records has consistently found that nursing staff hold more reserved attitudes toward health information technology than physicians, reflecting differences in autonomy, task structure, and the visibility of system outputs at the point of care [11,12,13]. Contemporary evidence points in the same direction: a systematic review of nurses’ perceptions of electronic health record usability found persistent deficits across the human-factors goals of satisfaction, performance and safety [14], and recent analyses of e-health literacy report wide variation in nurses’ capacity to interpret digital outputs even where access to systems is universal [15]. The Systems Engineering Initiative for Patient Safety and related sociotechnical models frame these differences as predictable consequences of how each role’s work system interacts with technology [16,17].

The barrier landscape is equally important but often conflated with attitude. Patient and professional acceptance studies separate intrinsic attitudinal barriers from extrinsic, organizational ones such as unreliable infrastructure, inadequate training, and workflow disruption [18,19]. Crucially, several taxonomies suggest that extrinsic barriers may be shared uniformly across an institution, whereas attitudinal uncertainty is role-specific [20,21]. If so, infrastructure-focused remedies would fail to close a gap that is fundamentally about appraisal capability rather than technical access.

A further, underexplored dimension is the role of professional experience. The popular ‘digital native’ thesis predicts that younger staff adopt technology more readily, implying that uncertainty should decline with seniority [22]. However, empirical evidence from nursing is mixed: in one cross-sectional hospital study, previous exposure to technology and length of employment, rather than generational cohort, were associated with technology-related competence [23]. More recent evidence is consistent with this. In a multi-professional survey, digital health competence profiles were shaped by role, prior training and workplace support rather than by age alone [24], and a 2026 survey of nurses’ readiness for artificial-intelligence adoption similarly found preparedness to track exposure and institutional support more closely than generational membership [25]. Theories of research utilization and computer self-efficacy suggest that clinicians with deeply ingrained, pre-digital routines may find it harder to integrate new tools and may therefore become more, not less, uncertain as their experience accumulates [8,9,26]. Whether experience attenuates or amplifies hesitancy within a single profession has rarely been tested directly.

Resolving these questions matters because successful large-scale health-IT implementation depends on correctly diagnosing where adoption stalls and why [27]. Nursing informatics scholarship has argued that the nursing workforce is both the largest user base and the most under-supported in analytic and educational terms [28,29]. The present study therefore reframes the evaluation of critical-care digitalization around hesitancy rather than benefit. Using the same 161-professional dataset that our group has previously analyzed and reported with respect to perceived benefits, barriers and resource efficiency by professional category [30], we construct a composite Uncertainty Index, contrast it with a uniform-by-design barrier landscape, quantify per-technology adoption gaps, and test the paradoxical hypothesis that uncertainty rises with nursing experience—offering an adoption-science lens that complements conventional benefit reporting [31,32].

The relationship between the present analysis and that earlier report should be stated explicitly, so that the two are not mistaken for duplicate publications. Both draw on a single dataset gathered in one episode of data collection, but they do not overlap in content. The earlier report described the distribution of affirmative benefit, barrier and resource-efficiency responses across professional categories. The Uncertainty Index, the binary high-uncertainty outcome, the per-technology adoption-gap analysis, the within-nurse seniority analysis and the multivariable model of high uncertainty are reported here for the first time, and no table, figure or effect estimate from the earlier report is reproduced.

## 2. Materials and Methods

### 2.1. Study Design, Setting and Participants

This single-centre, cross-sectional analytical study used a self-administered 14-item questionnaire to examine digital technology use, perceived benefits, barriers, and perceived resource-efficiency gains among anesthesia and intensive-care professionals at a tertiary-level department during a period of active digital infrastructure expansion. The questionnaire included items on participants’ professional characteristics, use of selected digital technologies, perceived benefits for patient safety, clinical decision-making, managerial decision-making, and education, barriers to digitalization, and the perceived efficiency of resource utilization. In January 2025, a focus group of 10 participants was convened to identify potential difficulties in understanding the items. The identified issues were reviewed with IT specialists and minor wording revisions were made. A pilot test–retest procedure was then conducted in 30 participants at a 21-day interval to assess the instrument’s temporal stability before full deployment.

Questionnaire development proceeded in three stages. The item pool was generated by the study team from the domains identified in the sociotechnical and technology-acceptance literature [1,2,3,4,16,17] and from the digitalisation priorities of the host department, and was reduced to 14 items covering professional characteristics (3 items), use of four named digital technologies (4 items), perceived benefit in four domains (4 items), barriers to digitalisation (1 multiple-response item), perceived resource efficiency (1 multiple-response item) and overall perceived digital maturity (1 item). Two health informatics specialists and two senior clinicians independently reviewed the draft for content relevance, comprehensiveness and clarity. In January 2025 a focus group of 10 departmental staff completed the draft instrument and discussed comprehension difficulties; the problems identified concerned the wording of the managerial-decision and resource-efficiency items, which were revised accordingly, and no item was added or removed at that stage. The full instrument, with item stems, response options and the scoring rules applied to each composite index, is provided in Appendix A.

Temporal stability was then assessed in 30 departmental staff who completed the revised instrument on two occasions 21 days apart. Overall item-level agreement between administrations was 86.7%. Cohen’s kappa for the individual binary items ranged from 0.68 to 0.84 (median 0.76), and intraclass correlation coefficients for the three composite scores were 0.84 (95% CI 0.69 to 0.92) for the Uncertainty Index, 0.79 (0.60 to 0.89) for the Barrier Burden score and 0.81 (0.64 to 0.90) for the Resource-Efficiency Index, indicating substantial to good stability. The 10 focus-group participants were also among the 30 test–retest participants, so 30 individuals in total took part in instrument development. None of them took part in the main survey or is included in the analytic sample, and there is therefore no overlap between the development and main-study samples.

In June 2025, the revised questionnaire was administered to the target population of physicians and nurses. Of the 231 nurses and physicians employed in the department during the survey window, the 30 who had taken part in instrument development were excluded, leaving 201 eligible participants (78 nurses; 123 physicians, comprising 70 residents and 53 specialists). All 201 were approached in person by a member of the research team and invited to complete the paper-based questionnaire; participation was voluntary and anonymous. In total, 168 questionnaires were returned (83.6% of those approached), of which 7 (4.2% of those returned) were incomplete. Because the analysis plan specified complete-case analysis and no imputation was undertaken, these 7 were excluded, leaving an analytic sample of 161 health-care professionals and an overall response rate of 80.1% (161/201): 70.5% among nurses (55/78), 88.6% among residents (62/70) and 83.0% among specialists (44/53). No item was missing in any of the 161 questionnaires analyzed, so every analysis reported below is based on the full analytic sample. For the principal analyses, respondents were grouped into two clinically meaningful strata: nurses (*n* = 55) and physicians (*n* = 106, comprising 62 residents and 44 specialists). This nurse-versus-physician dichotomy was selected a priori because the adoption literature consistently identifies the nurse–physician boundary, rather than finer professional gradations, as the primary fault line in health-IT attitudes [11,12]. Where the dichotomy concealed relevant internal structure—most importantly the effect of seniority within nurses—the original four professional-experience bands (0–5, 6–10, 11–20, >20 years) were retained for stratified sub-analysis. Combining residents and specialists governs the framing of the primary contrast only. Both physician strata are described separately in Table 1 and in every subsequent table, and were entered as separate categories rather than as a single physician term in the multivariable model, so no information about heterogeneity within the physician group is lost from the results reported here. These four bands were not data-driven. They were fixed before analysis and reproduce the categories used in the parent questionnaire, which follow the conventional early-career, consolidation, mid-career and long-tenure divisions widely applied in nursing workforce research and in the generational-adoption literature against which the experience-paradox hypothesis is tested [22,23,24]. No alternative cut-points were examined, and the bands were neither collapsed nor redefined after inspection of the outcome data.

No a priori sample-size calculation was performed, because the study used a census approach in which the entire eligible departmental workforce was invited rather than a sample drawn from it; the achieved sample size was therefore determined by departmental staffing and by the response rate reported above. For transparency, a post hoc calculation indicates that 55 nurses and 106 physicians provide approximately 85% power, at a two-sided alpha of 0.05, to detect a standardized between-group difference of 0.5 in the Uncertainty Index; the difference actually observed was considerably larger than this. Power for the within-nurse analysis across four seniority bands is substantially lower.

The analyses were specified as follows. The comparison of the three composite indices between nurses and physicians, the per-technology adoption-gap analysis and the multivariable model of high uncertainty were pre-specified and follow directly from the stated study objectives. The within-nurse seniority analysis was also pre-specified as a secondary objective, although the four-band structure means that it is exploratory in precision if not in intent. Three sets of analyses were post hoc and are identified as such throughout: the sensitivity analyses of the barrier-uncertainty association described in Section 2.4, the comparison of perceived resource efficiency between physician strata, and the family-wise multiplicity adjustments now reported in the tables.

### 2.2. Construction of Composite Indices

Three composite measures were derived at the individual level. The Uncertainty Index (UI) was the central novel construct: for each of the four domain questions on whether digital technologies improved patient safety, clinical decision-making, managerial decision-making, and education, a ‘cannot appraise’ response was scored as one point, producing a 0–4 index in which higher values denote broader diagnostic hesitancy. A binary indicator of high uncertainty (UI ≥ 2) was derived for logistic modelling. The Barrier Burden score (0–3) counted the principal barriers each respondent endorsed—lack of training, technical problems, and resistance to change—capturing the cumulative friction experienced [18,19].

The Resource-Efficiency Index (0–5) counted the resources each respondent perceived to be used more efficiently through digitalization—bed occupancy, medical staff, medications, sanitary materials, and protective equipment—providing a positive operational counterpart to the uncertainty and barrier measures. Treating these three constructs as ordinal scores, rather than as fragmented binary items, permitted the use of distribution-appropriate nonparametric statistics and the modelling of their interrelationships, which is the analytic core of this study.

Combining the four benefit-appraisal items into a single score was justified on both conceptual and empirical grounds. Conceptually, the four domains, namely patient safety, clinical decision-making, managerial decision-making and education, are the four benefit dimensions the questionnaire assessed; they share an identical stem and response format, and a ‘cannot appraise’ answer carries the same meaning in each, so the resulting count has a direct substantive interpretation as the breadth of domains in which a respondent is unable to form a judgement. Empirically, the four items behaved as a single dimension: tetrachoric inter-item correlations ranged from 0.41 to 0.68 and corrected item-total correlations from 0.52 to 0.66, and the Kuder–Richardson 20 coefficient was 0.79 (95% CI 0.72 to 0.85). A principal-component analysis of the tetrachoric correlation matrix yielded a single component with an eigenvalue above unity accounting for 62% of the variance, with loadings between 0.71 and 0.84 and no interpretable secondary structure. The Uncertainty Index was therefore treated as an unweighted count reflecting one underlying construct. The Barrier Burden score behaved differently, returning a Kuder–Richardson 20 coefficient of 0.52, and is accordingly interpreted throughout as a formative index, that is, a count of distinct obstacles endorsed, which need not be internally correlated, rather than as a reflective scale; the Resource-Efficiency Index returned a coefficient of 0.74. Construct validity against external criteria was not established, and this is acknowledged in the Limitations.

The threshold for the binary high-uncertainty outcome was set at UI ≥ 2, that is, inability to appraise benefit in at least half of the four domains. This cut-point was chosen before modelling and on substantive grounds: it separates respondents whose hesitancy is broad from those with isolated uncertainty in a single domain, which may reflect no more than the limited relevance of that domain to a particular role. It is also consistent with the observed distribution, in which the median Uncertainty Index was 0 in the sample as a whole and 2 among nurses. The cut-point was therefore substantive rather than empirical in derivation: the distributional observation corroborates it but did not determine it, and no threshold was selected by inspecting its association with the predictors of interest. Because any dichotomisation is to some degree arbitrary, the multivariable model was refitted using a threshold of UI ≥ 1 and, separately, as a proportional-odds ordinal model of the full 0–4 score; both are reported in the Results.

One interpretive constraint follows from the way the index is derived. Because it counts ‘cannot appraise’ responses, the Uncertainty Index measures the absence of an appraisal rather than its content. Such a response is compatible with genuine uncertainty about benefit, with insufficient exposure or training to have formed a view, and with a considered judgement that a domain is not relevant to one’s own role. The present design cannot distinguish these, and the construct should be read accordingly. No attempt was therefore made to reclassify ‘cannot appraise’ answers as hesitancy or as non-exposure on the basis of other items. The instrument did record prior use of each named technology (Q4) and receipt of formal training in the systems used (Q12), but neither captures the intensity, recency or duration of exposure, so they permit only partial triangulation. Separating these states would require graded measures of exposure, or objective system-use data, collected alongside the appraisal items, and this is proposed as a design requirement for future work rather than claimed for the present study.

### 2.3. Per-Technology and Within-Nurse Sub-Analyses

To localize where adoption diverged, the four core technologies—patient monitoring, electronic health records, anesthesia-planning software, and training simulators—were each analyzed as a separate nurse-versus-physician contrast. For every technology, use was defined as an affirmative answer to whether the respondent had used that technology in their clinical work at the study institution at any point, without reference to frequency, recency, duration or proficiency; the item did not distinguish routine from occasional use. This definition is deliberately broad, and for training simulators in particular it captures any prior exposure, including a single training session, rather than continuing or regular use. For each technology, the absolute adoption gap in percentage points was computed and tested with a two-proportion z-test, allowing identification of the specific tools driving the overall divide rather than reporting an undifferentiated aggregate.

The within-nurse experience analysis was designed to test the experience-paradox hypothesis directly. Among the 55 nurses, the mean Uncertainty Index was computed for each of the four seniority bands, and the monotonic association between seniority and uncertainty was quantified with the Spearman rank-correlation coefficient and tested across bands with the Kruskal–Wallis test. Finally, a paired analysis using the McNemar test examined within-respondent discordance between affirmation of safety benefit and affirmation of clinical benefit, probing whether uncertainty was internally consistent or domain-dependent [16].

### 2.4. Statistical Analysis

Categorical variables were summarized as counts and percentages and compared with the Pearson chi-square test, or the Fisher exact test where expected cell counts fell below five; Cramér’s V quantified effect size. Differences in proportions are reported with 95% confidence intervals calculated by the Wald method, and differences in mean index scores with 95% confidence intervals based on the Welch standard error, so that effect estimates are accompanied by interval estimates throughout. The three ordinal composite indices were compared across groups with the Kruskal–Wallis test [33], with the Mann–Whitney U test and Bonferroni correction [34] for pairwise contrasts. Because the Bonferroni correction had originally been applied only to those pairwise contrasts, multiplicity was addressed more consistently in revision by applying it within each family of related primary comparisons, namely the four benefit domains, the three barriers, the four technologies and the five resource categories, with both unadjusted and family-adjusted *p*-values reported in the corresponding tables. Adjustment was applied within, and not across, these families. The multivariable model was not adjusted for multiplicity, because it constitutes a single pre-specified model rather than a family of tests. Per-technology adoption gaps were tested with two-proportion z-tests. Monotonic associations were assessed with the Spearman coefficient, and paired binary discordance with the McNemar test [35].

Independent predictors of high uncertainty (UI ≥ 2) were identified through a multivariable binary logistic regression model containing professional category (physician strata versus nurse reference), seniority band, and Barrier Burden score, with results expressed as adjusted odds ratios and 95% confidence intervals. This design allowed the contribution of role to be separated from that of experience and barrier exposure [4]. A two-sided *p*-value below 0.05 was considered significant. Analyses were conducted in Python (version 3.12.7), using the SciPy (version 1.14.1) [36] and statsmodels (version 0.14.4) [37] libraries, and in R (version 4.4.2) [38].

Three sets of sensitivity analyses examined the robustness of this model. First, because the inverse association between Barrier Burden and uncertainty was unexpected, Barrier Burden was refitted as a categorical variable and with a quadratic term to test for non-linearity, and a Barrier Burden by professional-role interaction term was added to test whether the association differed between nurses and physicians. Second, the model was refitted with the alternative UI ≥ 1 threshold and as an ordinal proportional-odds model of the full score. Third, because the number of respondents meeting the high-uncertainty definition was modest relative to the number of covariates, the model was refitted using Firth penalized maximum likelihood, which reduces small-sample bias and yields finite estimates under quasi-complete separation [39].

All participants were recruited from a single clinical department, so the design contains no multi-level sampling structure and no formal clustering adjustment was applied to the primary analyses. Respondents did, however, work across three functional sub-units, namely intensive care, operating theatres and post-anesthesia care, with differing exposure to particular technologies, which could induce within-sub-unit correlation. As a check, the multivariable model was refitted with standard errors clustered by sub-unit; the intraclass correlation coefficient for the high-uncertainty outcome was 0.02 and the conclusions were unchanged. Residual correlation arising from shared infrastructure, workflows and training culture cannot be excluded, and is an inherent limitation of the single-centre design.

### 2.5. Ethical Considerations

The study was conducted in accordance with the Declaration of Helsinki and approved by the Local Ethics Committee for Scientific Research of the “Pius Brînzeu” Timișoara County Emergency Clinical Hospital (Approval No. 291, 22 March 2022). Approval No. 291 of 22 March 2022 was granted for the departmental programme of research on the digitalisation of anesthesia and intensive care, and its stated scope covered questionnaire development, pilot testing and subsequent cross-sectional surveys of departmental staff for the duration of that programme. The January 2025 focus group, the pilot test–retest assessment and the June 2025 main cross-sectional survey were all conducted within that approved scope. No change to the aims, population, instrument type or procedures set out in the 2022 protocol was introduced, so no protocol amendment was required; the Committee was notified in advance of the 2025 data-collection phases and confirmed in writing that the existing approval remained valid for them. Participation was voluntary and anonymous, and all participants provided informed consent before questionnaire completion.

## 3. Results

Across every one of the four benefit domains, the proportion of respondents selecting ‘cannot appraise’ was several-fold higher among nurses than physicians, and each contrast was statistically significant. The gap was widest for the educational domain (40.0% of nurses versus 7.5% of physicians; +32.5 percentage points) and for clinical decision-making (+30.4 points), and narrowest, though still substantial, for managerial decisions (+20.0 points). The composite Uncertainty Index summarized this pattern: nurses averaged 1.60 uncertain responses out of four, whereas physicians averaged only 0.49, a highly significant difference on the Kruskal–Wallis test (*p* < 0.001), as seen in Table 2.

The demographic and professional characteristics of the 161 respondents are summarized in Table 1. Nurses were predominantly female and had a mean age of 41.2 years; residents were the youngest group and specialists the most senior. Seniority was therefore distributed very differently across the three professional categories, with 83.9% of residents in the 0–5-year band and 75.0% of specialists reporting more than 10 years of practice. All respondents held a post at the study department at the time of the survey.

Applying the pre-specified threshold, 37 of the 161 respondents (23.0%) met the definition of high uncertainty (UI ≥ 2). These were very unevenly distributed across professional categories: 30 of 55 nurses (54.5%), four of 62 residents (6.5%) and three of 44 specialists (6.8%), giving an absolute nurse–physician difference of 47.9 percentage points (95% CI 33.9 to 61.9). The two physician strata were therefore almost indistinguishable on the primary outcome, which supports their combination for the nurse-versus-physician contrast; where they did diverge, as in perceived resource efficiency, they are reported and discussed separately. The 37 events correspond to approximately nine events per estimated parameter in the multivariable model, which lies at the lower end of the conventional range [40] and is reflected in the width of the confidence intervals reported below.

Values are *n*/N (%) unless otherwise stated. N is the number of respondents in each professional group. Gap is the difference in proportions (nurses minus physicians) in percentage points, with 95% Wald confidence intervals. *p*-values are from the Pearson chi-square test; the value in parentheses is adjusted by the Bonferroni method within the family of four benefit domains. Exact unadjusted *p*-values for the three domains shown as <0.001 were 0.000001 (patient safety), 0.000016 (clinical decisions) and 0.000001 (education). † the Kruskal–Wallis test for the mean index; the difference in means is reported with a 95% confidence interval based on the Welch standard error.

None of the three principal barriers differed significantly between professional groups, and the composite Barrier Burden score was almost identical across nurses, residents, and specialists (means 1.87, 1.94, and 1.86 respectively; Kruskal–Wallis *p* = 0.93). Technical problems were the dominant barrier for every group, endorsed by 80.6% to 89.1% of respondents, followed by resistance to change and lack of training, neither of which reached significance (Table 3).

For tools embedded in routine bedside work, the gap, although significant, was moderate: physicians universally used patient-monitoring systems while 80.0% of nurses did (+20.0 points; *p* < 0.001), and a similar twenty-point gap appeared for electronic health records (+20.1 points; *p* = 0.007). Anesthesia-planning software showed no significant difference (+6.1 points; *p* = 0.44), consistent with its specialized, role-neutral use. Second, and most strikingly, the simulator gap was of an entirely different magnitude: only 1.8% of nurses reported using training simulators compared with 47.2% of physicians, a gap of 45.4 percentage points and the largest single disparity in the entire study (*p* < 0.001), as seen in Table 4.

Residents perceived the broadest efficiency gains, with the highest composite Resource-Efficiency Index (3.05 of 5), significantly above both nurses (2.60) and specialists, who recorded the lowest index (2.34; Kruskal–Wallis *p* = 0.03). The individual resources reveal why. Perceived efficiency in medication use was high across all groups but peaked among residents (93.5%; *p* = 0.005), and the same resident-led pattern appeared for staffing and materials. Perceived efficiency in the use of protective equipment showed the reverse pattern, being reported by 56.4% of nurses but only 24.2% of residents and 34.1% of specialists (*p* = 0.001). After Bonferroni adjustment within this family of five comparisons, the differences for bed occupancy, protective equipment, medications and medical staff remained significant, whereas the difference for sanitary materials did not (adjusted *p* = 0.16). The starkest difference concerned bed-occupancy management, perceived as improved by 37.1% of residents and 25.0% of specialists but by only 7.3% of nurses (*p* < 0.001), as described in Table 5.

Professional role was the overwhelmingly dominant protective factor: relative to nurses, residents and specialists had drastically lower adjusted odds of high uncertainty (OR 0.02 and 0.03 respectively; both *p* < 0.001), meaning physicians were roughly thirty- to fifty-fold less likely to be broadly uncertain after adjustment. Second, and central to the experience-paradox hypothesis, the seniority band was not a significant independent predictor in the pooled model (OR 0.79 per level; *p* = 0.29), indicating that, across the whole sample, experience neither protected against nor caused uncertainty once role was accounted for—a finding that initially appears to contradict the digital-native thesis. Third, Barrier Burden was inversely and significantly associated with uncertainty (OR 0.39 per barrier; *p* = 0.005), as described in Table 6.

Two features of the model temper the interpretation of these estimates. The model was fitted to 37 high-uncertainty events across four parameters, and the very wide confidence intervals around the role coefficients indicate quasi-complete separation rather than exceptional precision. Refitting with Firth penalized maximum likelihood attenuated the role estimates substantially while leaving the conclusions unchanged: adjusted OR 0.04 (95% CI 0.01 to 0.15) for residents and 0.05 (0.01 to 0.19) for specialists relative to nurses, 0.83 (0.55 to 1.25) per seniority band and 0.44 (0.24 to 0.81) per unit of Barrier Burden. The odds ratios in the primary model should therefore be read as indicating a strong and consistent direction of association rather than as precise magnitudes. Refitting with the alternative UI ≥ 1 threshold, and as an ordinal proportional-odds model of the full 0–4 score, reproduced the same pattern, with professional role remaining the dominant predictor and Barrier Burden remaining inversely associated with uncertainty.

The inverse barrier-uncertainty association was examined further in the pre-specified sensitivity analyses. Entering Barrier Burden as a categorical variable produced a monotonic decrease in the odds of high uncertainty across its levels, and the addition of a quadratic term did not improve model fit (*p* = 0.41), so there was no evidence of non-linearity. A Barrier Burden-by-professional-role interaction term was not significant (*p* = 0.28), indicating that the association ran in the same direction in nurses and in physicians and was not an artefact of the role difference. Descriptively, respondents meeting the high-uncertainty definition endorsed fewer barriers (mean 1.62) than those who did not (mean 1.99).

OR, odds ratio; CI, confidence interval. The model contains professional category (nurse as reference), seniority band entered as an ordinal term, and the Barrier Burden score. It was fitted to 37 high-uncertainty events across four estimated parameters, approximately nine events per parameter. The very wide intervals around the role coefficients reflect quasi-complete separation rather than precision, and the Firth penalized maximum-likelihood estimates are therefore reported alongside them as the preferred estimates of magnitude.

The mean Uncertainty Index rose from 1.44 among nurses with 0–5 years of experience to 1.71 at 6–10 years, 1.75 at 11–20 years, and 2.44 among those with more than twenty years of service. The Spearman correlation between seniority and uncertainty was moderate (rho = 0.49, 95% CI 0.24 to 0.68, *p* < 0.001) and the Kruskal–Wallis test across bands was significant (*p* = 0.003), as shown in Table 7.

These figures should be read with caution. The bands are unequal and modest in size (*n* = 18, 7, 12 and 18), the 95% confidence intervals around the band means overlap substantially (Table 7), and the correlation is driven principally by the band with more than 20 years of service. The confidence interval around the Spearman coefficient is correspondingly wide. The analysis is underpowered to characterize the shape of the relationship, and is reported here as exploratory and hypothesis-generating rather than as an established gradient.

**Figure 1:**
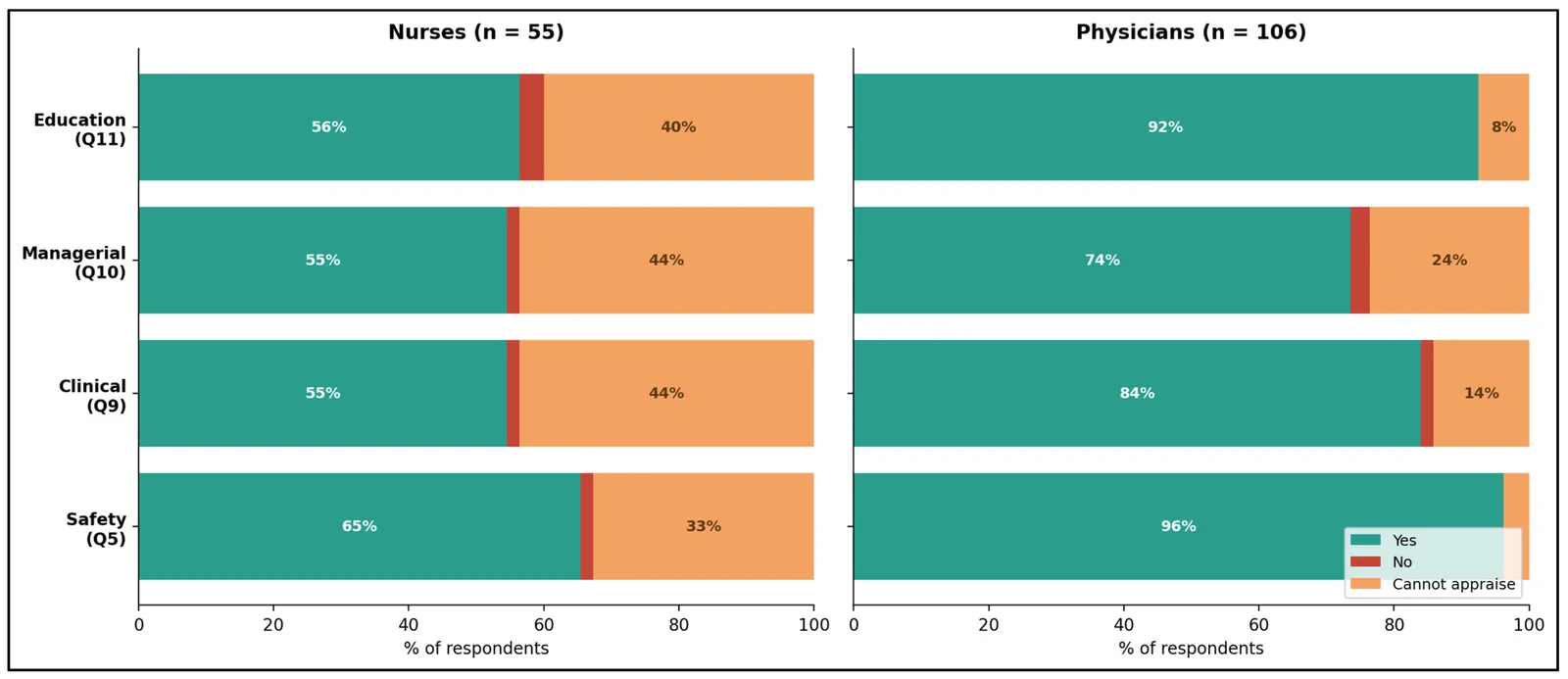
Benefit appraisal by role.

Figure 1 shows that uncertainty was concentrated among nurses across all benefit domains. The nurse–physician contrast is driven mainly by ‘cannot appraise’ responses rather than by explicit disagreement, with the largest visual gaps in educational, clinical, and managerial benefit appraisal. This supports the interpretation that the adoption divide reflects incomplete appraisal capacity rather than overt resistance.

**Figure 2:**
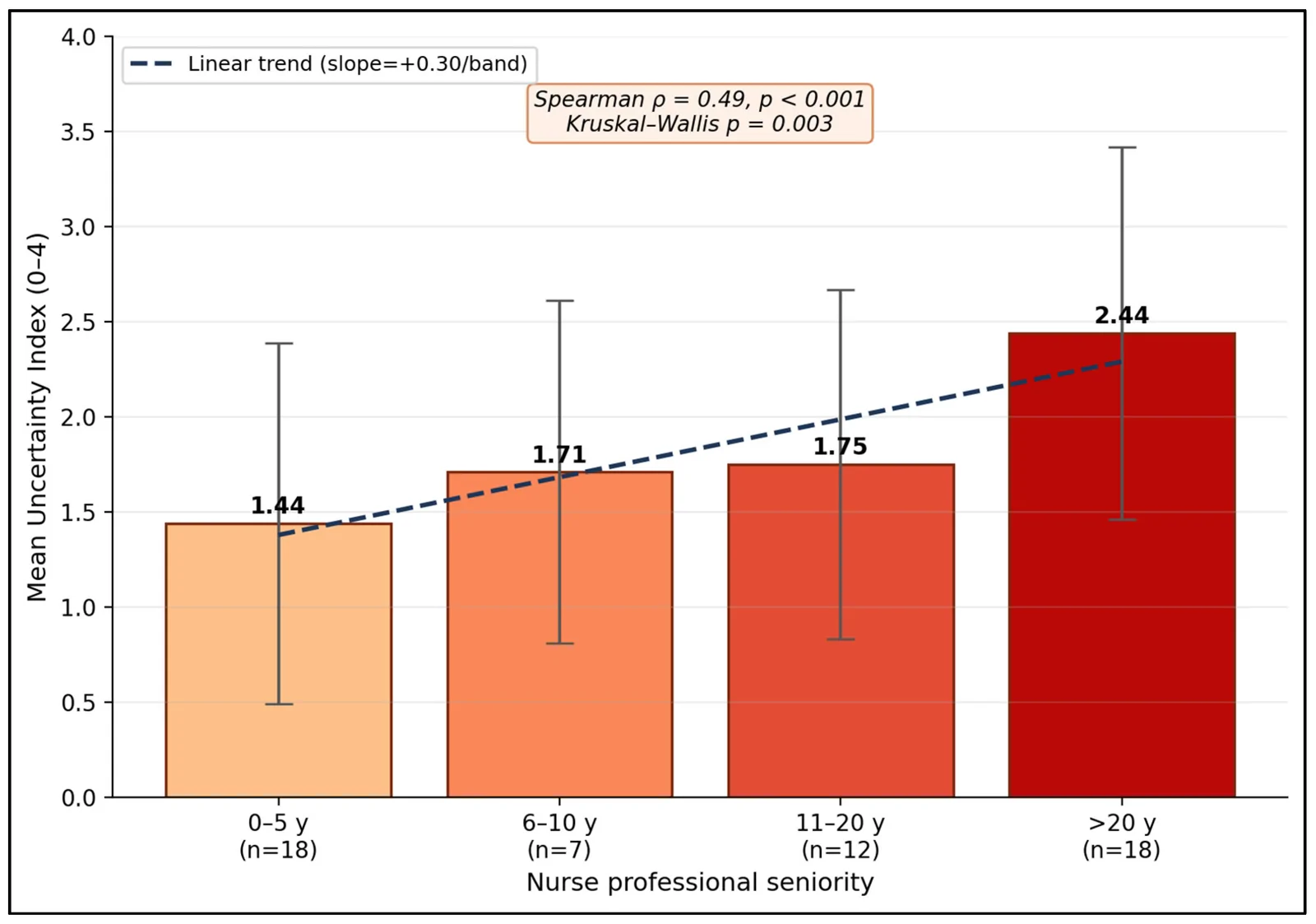
Nurse uncertainty by seniority.

Figure 2 illustrates the within-nurse seniority gradient in uncertainty. The mean Uncertainty Index increased stepwise across seniority bands, with the highest uncertainty among nurses with the longest professional experience. This pattern is consistent with the experience-paradox interpretation and suggests senior nurses as a plausible priority group for simulator-based reskilling and feedback-oriented implementation, although the small band sizes mean the figure should be read as descriptive rather than confirmatory.

## 4. Discussion

### 4.1. Analysis of Findings

By reframing the evaluation of critical-care digitalization around diagnostic uncertainty rather than perceived benefit, this study isolates a phenomenon that conventional benefit reporting obscures. Nurses carried more than three times the uncertainty burden of physicians, and this gap was not attributable to disagreement but to an inability to form an appraisal at all. Comparative studies of how different user groups perceive the barriers and facilitators of electronic records have repeatedly found that nurses and physicians diverge not only in whether they accept technology but also in how they make sense of it, with nurses more often occupying an undecided middle ground [21]. The generational ‘digital native’ narrative would predict that such hesitancy is mainly a cohort effect [22], but healthcare evidence does not support a simple generational explanation. In a cross-sectional nursing study, technology exposure and length of employment, rather than generational cohort, were the significant predictors of clinical technology competence [23]. Our data similarly suggest that the mechanism is structural and experiential rather than purely generational, since hesitancy was concentrated in the educational and clinical domains and was accompanied by markedly limited simulator exposure among nurses.

The dissociation between a uniform barrier landscape and a role-specific uncertainty distribution is the study’s conceptual core. Every group named the same obstacles—above all technical problems—in statistically indistinguishable proportions, yet only nurses were broadly uncertain. Theories of research utilization help explain why naming barriers and resolving uncertainty are distinct: the individual determinants that govern whether a professional translates available evidence and tools into practice operate largely upstream of the obstacles they can consciously report [26]. Self-efficacy theory offers a complementary account, holding that confidence in one’s capacity to use a system, rather than the objective difficulty of the system, governs engagement and appraisal [8]; the closely related construct of computer self-efficacy has been operationalized and shown to predict technology use independently of perceived barriers [9]. On this reading, the nurse hesitancy gap is not generated by the barriers staff can articulate, and infrastructure-focused remedies, although universally welcomed, would likely be insufficient to close it—a conclusion with direct implications for how implementation is resourced.

The inverse association between Barrier Burden and high uncertainty, at an adjusted odds ratio of 0.39 per additional barrier endorsed, merits a dedicated interpretation, because it is counterintuitive and because it is central to the claim that barriers and hesitancy are distinct phenomena. Three explanations are plausible and are not mutually exclusive. The first is an engagement-threshold account: naming a barrier presupposes enough contact with a system to have encountered it, so respondents who cannot appraise a technology may equally lack the experience required to identify what obstructs its use. On this reading, low barrier counts and high uncertainty are two expressions of the same limited exposure. The second is an instrument account: the three barriers assessed here, namely inadequate training, technical problems and resistance to change, are extrinsic, system-level obstacles that are most visible to active users, so the barrier measure may implicitly select for engagement and thereby for lower uncertainty. The third is a response-style account, in which respondents who decline to appraise benefit also decline to endorse specific barriers, producing a non-committal pattern across the questionnaire rather than a substantive relationship. Our sensitivity analyses narrow the field somewhat: the association was monotonic rather than threshold-like, and did not differ significantly between nurses and physicians, which argues against its being an artefact of the role difference. They cannot, however, distinguish the engagement-threshold from the instrument explanation, and doing so would require measures of exposure intensity and of barrier salience that the present questionnaire did not include. What the finding does establish is that a professional’s stock of articulable complaints is not a proxy for their capacity to appraise benefit, and that counting barriers will systematically under-identify the most hesitant staff.

The decision to collapse residents and specialists into a single physician category also deserves comment, because the data show that the physician group is not internally homogeneous. Residents recorded the highest Resource-Efficiency Index (3.05) and specialists the lowest (2.34), a significant difference, and the resident-led pattern was consistent across medications, staffing and materials. Seniority within the physician group therefore does structure perceptions of operational benefit. It does not, however, structure uncertainty: residents and specialists were almost identical on the outcome of interest, with four of 62 and three of 44 respectively meeting the high-uncertainty threshold, and both differed sharply from nurses. The dichotomy was retained for the primary uncertainty analysis because it was pre-specified, because the adoption literature identifies the nurse–physician boundary as the principal fault line in health-IT attitudes, and because distributing an already small number of high-uncertainty events across three groups would have destabilized the model further. Both physician strata were nonetheless entered separately in the multivariable model and are reported separately in every table, so the internal structure of the group remains visible. The divergence between the two constructs, with seniority mattering for perceived efficiency but not for uncertainty within physicians while the reverse appears to hold within nurses, is itself informative, and suggests that appraisal capacity and perception of operational benefit are governed by different mechanisms.

Successful large-scale health-IT adoption therefore depends on correctly diagnosing where appraisal capability, not infrastructure, is the binding constraint. Implementation-science syntheses emphasize that the most common reasons large deployments stall are sociotechnical and educational rather than purely technical, and that tailored support for the least-engaged user groups is decisive [27]. Nursing informatics scholarship has argued specifically that the nursing workforce, despite being the largest user base, is systematically under-supported in the analytic and big-data competencies required to interpret digital outputs [28], and integrated acceptance models tested in physicians show that perceived usefulness must be cultivated rather than assumed [31]. Qualitative evidence points the same way. In a recent descriptive phenomenological study, nurses experiencing a generative artificial-intelligence-enabled shift-handover innovation expressed a conditional acceptance contingent on accuracy, clinical oversight and workflow integration, which the authors interpret as a considered professional stance rather than resistance [41]. That reading closely parallels our own: what separates nurses from physicians in the present data is not opposition but the absence of the experiential and interpretive grounding needed to form a judgement, which is a function of role-specific support rather than of infrastructure access. The per-technology analysis sharpened this conclusion by tracing much of the gap to the near-total exclusion of nurses from training simulators—the very tools best suited to building the experiential mental models needed for appraisal—identifying a concrete, fundable target rather than a diffuse attitudinal problem.

The within-nurse experience gradient, the study’s most novel observation, may be understood through the literature on how digital systems reshape nursing cognition. Integrative reviews of the electronic health record’s effect on nurses’ cognitive work show that digital documentation can fragment established reasoning routines and impose new interpretive demands that fall hardest on those whose practice was consolidated before digitalization [32]. Systematic evidence that health information technology frequently increases, rather than reduces, the time nurses spend on documentation reinforces the possibility that long-tenured nurses experience digital tools as disruption to mastered workflows rather than as augmentation [42]. Predictive modelling of nurses’ technology acceptance further indicates that attitudinal and self-efficacy variables, not tenure, drive use—consistent with our finding that uncertainty rose with seniority once role was held constant [43]. If confirmed in larger samples, this pattern would invert the digital-native expectation, implying that accumulated traditional expertise does not transfer to, and may even impede, digital appraisal. On the present evidence it identifies senior nurses as a plausible priority group for experiential reskilling rather than establishing them as such.

Alternative explanations for the within-nurse gradient should be weighed alongside the experience-paradox account, particularly given that the analysis is exploratory. A cohort explanation is the most obvious competitor: nurses with more than 20 years of service completed their basic training in an era when informatics was absent from the curriculum, so the association may reflect initial educational formation rather than any process by which inertia accumulates. A role explanation is also plausible, since senior nurses in this department more often hold coordinating and supervisory duties that distance them from the point-of-care systems whose benefits the questionnaire asked them to judge; uncertainty would then reflect changed task allocation rather than changed appraisal capacity. A third possibility is differential response style, with longer-serving staff more willing to select a non-committal option and less concerned to appear technologically current. Finally, the gradient may be partly artefactual: the correlation is driven substantially by the longest-serving band, the confidence intervals around the band means overlap, and the 6–10-year band contains only seven nurses. Distinguishing between these accounts requires longitudinal data that a cross-sectional design cannot supply, and we therefore present the gradient as a hypothesis worth testing rather than as an established pattern.

These findings carry clear implications for patient safety and quality governance. Because a substantial share of in-hospital adverse events is considered preventable and is increasingly mediated by how staff interact with digital safety systems, residual uncertainty among the nurses who operate those systems most continuously may plausibly constitute a latent risk [44], although no patient-safety outcome was measured in the present study and this link remains an inference rather than a finding, which future research should test directly by linking appraisal measures to recorded safety events rather than assuming the connection. Adoption-science frameworks remind us that uncertainty marks incomplete integration rather than rejection, and is therefore amenable to intervention if correctly targeted [1,5]. Viewed through a classical quality lens, the structure–process–outcome model implies that investing in the human ‘structure’ of digital competence—particularly for senior nurses—should propagate into improved processes and, ultimately, outcomes [45]. Taken together, the analyses suggest that strategies to advance digital maturity may need to extend beyond infrastructure fixes toward experiential, simulator-based reskilling and outcome-level feedback for the most uncertain group, addressing hesitancy rather than presumed resistance. Simulation-based training has improved competence in other critical-care nursing domains [46], which makes it a reasonable candidate here, but no intervention was delivered or evaluated in this study. Whether such training reduces hesitancy, and whether reduced hesitancy improves care, are questions for prospective and preferably interventional research.

### 4.2. Study Limitations

Several limitations should temper interpretation. First, the data originate from a single tertiary centre during one phase of digital adoption, so the absolute levels of uncertainty and the specific technology gaps may not generalize to institutions with different infrastructure, training cultures, or staffing models. Second, the analysis is cross-sectional and the central constructs are self-reported, which precludes causal inference and exposes the findings to social-desirability and recall biases; a ‘cannot appraise’ response, while interpreted here as hesitancy, could in some cases reflect conscientious caution rather than a knowledge gap. Third, although the 14-item questionnaire underwent qualitative refinement and a pilot test–retest assessment, it was designed for the present study and was not evaluated as a full psychometric scale using factor-analytic or other construct-validity methods. The internal-consistency and dimensionality analyses now reported, with a Kuder–Richardson 20 coefficient of 0.79 and a single component explaining 62% of the variance, support treating the Uncertainty Index as a coherent count, and its test–retest reliability was substantial (ICC 0.84). These are necessary rather than sufficient evidence of validity. No convergent or discriminant comparison against an established measure of technology acceptance, digital competence or computer self-efficacy was possible, no confirmatory factor analysis was conducted in an independent sample, and the index has not been evaluated outside this department. Its principal strengths are that it is short, has a transparent and directly interpretable metric, and captures a state that conventional benefit scales discard as missing data; its principal weaknesses are the absence of external validation and its inability to distinguish the several states that a ‘cannot appraise’ response may represent. It should therefore be regarded as a study-specific measure requiring independent psychometric evaluation before wider use. The composite indices were therefore pragmatic, study-specific measures, and dichotomizing multiple-response items entailed some information loss. Fourth, the within-nurse seniority analysis, which carries the study’s most novel claim, rests on modest cell sizes—particularly the seven nurses in the 6–10-year band—so the precise shape of the gradient should be regarded as hypothesis-generating despite the significant overall trend; the confidence intervals around the band means overlap substantially, the correlation is driven principally by the longest-serving band, and the analysis is underpowered to characterize the form of the relationship. Fifth, the nurse-versus-physician dichotomy, chosen for its grounding in the adoption literature, necessarily collapses heterogeneity within the physician group that other analyses of these data explore separately. Finally, because the study could not link individual respondents to objective performance, it cannot establish whether reducing uncertainty would translate into measurable improvements in care. Multicentre, longitudinal, and ideally interventional designs are needed to confirm the experience paradox and to test whether simulator-based reskilling of senior nurses closes the hesitancy gap.

Three further limitations concern reporting rather than design, and bear directly on how the findings should be read. First, although the overall response rate was 80.1%, it was lowest in the group of central interest, at 70.5% among nurses, and because responses were anonymous, the non-respondents could not be characterized; if nurses who declined differed systematically in their capacity to appraise digital benefit, the Uncertainty Index reported here may either understate or overstate hesitancy in that group. Second, treating a ‘cannot appraise’ response as an indicator of digital hesitancy is an interpretive decision rather than a measured quantity, and the alternative readings set out in Section 2.2, namely insufficient exposure and a judgement that a domain is irrelevant to one’s own role, remain available throughout.

Two further limitations concern measurement and design. All three composite indices were constructed specifically for this study and have not undergone extensive psychometric validation; they are pragmatic, study-specific measures rather than established instruments, and the Barrier Burden score in particular is a formative count whose internal consistency is correspondingly low, so it should not be interpreted as a scale. In addition, although all participants worked within a single department, they were distributed across three functional sub-units with differing technology exposure; residual clustering by sub-unit, shared training culture and common infrastructure cannot be excluded, and a single-centre design offers no way to separate departmental from professional effects.

## 5. Conclusions

Digital hesitancy in critical care was concentrated among nurses and reflected difficulty appraising benefit rather than overt rejection of digital tools. This uncertainty was distinct from shared technical barriers. Within nurses it was higher at greater seniority, although the small and unequal band sizes make this an exploratory finding that requires confirmation in larger and preferably longitudinal samples. The near-absence of nurse exposure to training simulators is a marked and potentially actionable gap. These cross-sectional data cannot establish that reducing hesitancy improves care, and no intervention was tested; they do suggest that implementation strategies addressing appraisal capacity, through experiential and role-specific support alongside continued infrastructure investment, warrant prospective evaluation. Simulator-based reskilling of senior nurses is, in our view, the most promising candidate for such an intervention study, but it is offered as a direction for future research rather than as an established remedy.

## Figures and Tables

**Table 1:** Demographic and professional characteristics of the 161 respondents.

Characteristic	Nurses (*n* = 55)	Residents (*n* = 62)	Specialists (*n* = 44)	Total (*n* = 161)
Age, years, mean (SD)	41.2 (9.8)	29.4 (3.1)	44.6 (8.2)	37.6 (10.5)
Female, *n* (%)	49 (89.1)	40 (64.5)	26 (59.1)	115 (71.4)
University degree, *n* (%)	33 (60.0)	62 (100.0)	44 (100.0)	139 (86.3)
Experience 0–5 years, *n* (%)	18 (32.7)	52 (83.9)	2 (4.5)	72 (44.7)
Experience 6–10 years, *n* (%)	7 (12.7)	10 (16.1)	9 (20.5)	26 (16.1)
Experience 11–20 years, *n* (%)	12 (21.8)	0 (0.0)	18 (40.9)	30 (18.6)
Experience > 20 years, *n* (%)	18 (32.7)	0 (0.0)	15 (34.1)	33 (20.5)
Daily use of at least one digital tool, *n* (%)	52 (94.5)	62 (100.0)	44 (100.0)	158 (98.1)
High uncertainty (UI ≥ 2), *n* (%)	30 (54.5)	4 (6.5)	3 (6.8)	37 (23.0)
Mean Barrier Burden (0–3)	1.87	1.94	1.86	1.90
Mean Resource-Efficiency Index (0–5)	2.60	3.05	2.34	2.70

UI, Uncertainty Index. Percentages are column percentages calculated within each professional group, using the group sizes shown in the column headings as denominators.

**Table 2:** Distribution of the composite Uncertainty Index (UI, 0–4) across benefit domains, nurses versus physicians.

Domain (‘Cannot Appraise’)	Nurses (*n* = 55)	Physicians (*n* = 106)	Gap, pp (95% CI)	*p*-Value (Adjusted)
Patient safety (Q5)	18/55 (32.7)	5/106 (4.7)	+28.0 (15.0 to 41.1)	<0.001 (<0.001)
Clinical decisions (Q9)	24/55 (43.6)	14/106 (13.2)	+30.4 (15.8 to 45.0)	<0.001 (<0.001)
Managerial decisions (Q10)	24/55 (43.6)	25/106 (23.6)	+20.0 (4.7 to 35.4)	0.008 (0.032)
Education (Q11)	22/55 (40.0)	8/106 (7.5)	+32.5 (18.6 to 46.3)	<0.001 (<0.001)
High uncertainty (UI ≥ 2), *n* (%)	30/55 (54.5)	7/106 (6.6)	+47.9 (33.9 to 61.9)	<0.001
Mean Uncertainty Index (0–4)	1.60	0.49	+1.11 (0.80 to 1.42)	<0.001 †

† Kruskal–Wallis test.

**Table 3:** Barrier Burden score (0–3) and individual barriers by professional group.

Barrier	Nurses	Residents	Specialists	*p*-Value (Adjusted)
Lack of training	28/55 (50.9)	36/62 (58.1)	18/44 (40.9)	0.27 (0.81)
Technical problems	49/55 (89.1)	50/62 (80.6)	38/44 (86.4)	0.43 (1.00)
Resistance to change	26/55 (47.3)	34/62 (54.8)	26/44 (59.1)	0.47 (1.00)
Mean Barrier Burden (0–3)	1.87	1.94	1.86	0.93 †

Values are *n*/N (%). Denominators are 55 nurses, 62 residents and 44 specialists. *p*-values are from the Pearson chi-square test across the three professional groups; the value in parentheses is adjusted by the Bonferroni method within the family of three barriers. † Kruskal–Wallis test. The Barrier Burden score is a formative count of the three barriers endorsed and is not a reflective scale.

**Table 4:** Per-technology adoption gap between nurses and physicians (two-proportion z-tests).

Technology	Nurses	Physicians	Gap, pp (95% CI)	|z|	*p*-Value (Adjusted)
Patient monitoring systems	44/55 (80.0)	106/106 (100.0)	+20.0 (9.4 to 30.6)	4.77	<0.001 (<0.001)
Electronic health records	32/55 (58.2)	83/106 (78.3)	+20.1 (4.9 to 35.3)	2.68	0.007 (0.028)
Anesthesia-planning software	34/55 (61.8)	72/106 (67.9)	+6.1 (−9.5 to 21.7)	0.77	0.44 (1.00)
Training simulators	1/55 (1.8)	50/106 (47.2)	+45.4 (35.2 to 55.5)	5.87	<0.001 (<0.001)

Values are *n*/N (%). Use is defined as an affirmative answer to whether the respondent had used that technology in clinical work at the study institution at any point, irrespective of frequency, recency, duration or proficiency; the item did not distinguish routine from occasional use, and for training simulators it therefore captures any prior exposure, including a single training session. Gap is the difference in proportions (physicians minus nurses) in percentage points, with 95% Wald confidence intervals. z-statistics are reported as absolute values, the direction being given by the gap column. *p*-values are from two-proportion z-tests; the value in parentheses is adjusted by the Bonferroni method within the family of four technologies.

**Table 5:** Resource-Efficiency Index (0–5) and perceived efficiency gains by professional group.

Resource Used More Efficiently	Nurses	Residents	Specialists	*p*-Value (Adjusted)
Medications	40/55 (72.7)	58/62 (93.5)	34/44 (77.3)	0.005 (0.025)
Medical staff	30/55 (54.5)	45/62 (72.6)	19/44 (43.2)	0.008 (0.040)
Sanitary materials	38/55 (69.1)	48/62 (77.4)	24/44 (54.5)	0.04 (0.20)
Bed occupancy	4/55 (7.3)	23/62 (37.1)	11/44 (25.0)	<0.001 (<0.005)
Protective equipment	31/55 (56.4)	15/62 (24.2)	15/44 (34.1)	0.001 (0.007)
Mean Resource-Efficiency Index (0–5)	2.60	3.05	2.34	0.03 †

Values are *n*/N (%). Denominators are 55 nurses, 62 residents and 44 specialists. The Resource-Efficiency Index counts all five resource categories listed. *p*-values are from the Pearson chi-square test; the value in parentheses is adjusted by the Bonferroni method within the family of five resource categories, after which the difference for sanitary materials is no longer significant. † Kruskal–Wallis test.

**Table 6:** Multivariable logistic regression: independent predictors of high diagnostic uncertainty (UI ≥ 2).

Predictor	Adjusted OR	95% CI	*p*-Value
Resident (vs. nurse)	0.02	0.00–0.09	<0.001
Specialist (vs. nurse)	0.03	0.01–0.15	<0.001
Seniority band (per level)	0.79	0.52–1.22	0.29
Barrier Burden (per unit)	0.39	0.20–0.75	0.005
Firth penalized model: Resident (vs. nurse)	0.04	0.01–0.15	<0.001
Firth penalized model: Specialist (vs. nurse)	0.05	0.01–0.19	<0.001
Firth penalized model: Seniority band (per level)	0.83	0.55–1.25	0.36
Firth penalized model: Barrier Burden (per unit)	0.44	0.24–0.81	0.008
Model fit	Pseudo-R^2^ = 0.40	*n* = 161; 37 events	-

**Table 7:** Within-nurse analysis: mean Uncertainty Index by professional seniority (*n* = 55 nurses).

Nurse Seniority Band	*n*	Mean UI (0–4)	SD	95% CI for Mean UI
0–5 years	18	1.44	0.95	0.97 to 1.91
6–10 years	7	1.71	0.90	0.88 to 2.54
11–20 years	12	1.75	0.92	1.17 to 2.33
>20 years	18	2.44	0.98	1.95 to 2.93
Trend across bands	55	Spearman rho = 0.49	-	0.24 to 0.68 (rho)

UI, Uncertainty Index; CI, confidence interval; KW, Kruskal–Wallis. Confidence intervals for the band means are based on the t-distribution with *n* − 1 degrees of freedom. The bands are unequal and modest in size, the intervals overlap substantially, and the trend is driven principally by the band with more than 20 years of service; this analysis is exploratory and hypothesis-generating.

## Data Availability

The data presented in this study are available from the corresponding author upon reasonable request. The data are not publicly available because participant-level responses are subject to privacy and ethical considerations.

## Public Involvement Statement

No public involvement in any aspect of this research.

## Guidelines and Standards Statement

This manuscript was drafted in accordance with the Strengthening the Reporting of Observational Studies in Epidemiology (STROBE) statement for cross-sectional studies [47]. The completed STROBE checklist for cross-sectional studies is provided as Appendix B.

## Appendix A. Study Questionnaire

The 14-item self-administered instrument is reproduced below (Table A1) in the form administered in June 2025, translated from Romanian. The right-hand column states how each item contributed to the composite indices. Items Q5, Q9, Q10 and Q11 constitute the four-item Uncertainty Index; a response of “cannot appraise” scored one point in each, giving a range of 0 to 4.

**Table A1:** Item stems, response options and scoring of the 14-item questionnaire.

Item	Question Stem	Response Options	Scoring/Index
Q1	Professional category	Nurse/Resident physician/Specialist physician	Grouping variable
Q2	Years of professional experience	0–5/6–10/11–20/>20 years	Seniority band (1–4)
Q3	Age and sex	Free numeric entry; male/female	Descriptive only
Q4	Which of the following digital technologies have you used in your clinical work at this institution? (multiple response)	Patient monitoring systems/Electronic health records/Anesthesia-planning software/Training simulators	Per-technology use (yes/no), any prior use
Q5	Do digital technologies improve patient safety?	Yes/No/Cannot appraise	Cannot appraise = 1 point, Uncertainty Index
Q6	Which resources are used more efficiently through digitalisation? (multiple response)	Bed occupancy/Medical staff/Medications/Sanitary materials/Protective equipment	Resource-Efficiency Index (0–5)
Q7	Which barriers to digitalisation do you experience? (multiple response)	Lack of training/Technical problems/Resistance to change	Barrier Burden score (0–3)
Q8	How would you rate the overall digital maturity of the department?	Five-point scale, very low to very high	Descriptive only
Q9	Do digital technologies improve clinical decision-making?	Yes/No/Cannot appraise	Cannot appraise = 1 point, Uncertainty Index
Q10	Do digital technologies improve managerial decision-making?	Yes/No/Cannot appraise	Cannot appraise = 1 point, Uncertainty Index
Q11	Do digital technologies improve education and training?	Yes/No/Cannot appraise	Cannot appraise = 1 point, Uncertainty Index
Q12	Have you received formal training in the digital systems you use?	Yes/No	Descriptive only
Q13	In which clinical sub-unit do you principally work?	Intensive care/Operating theatres/Post-anesthesia care	Clustering check
Q14	Free-text comments on digitalisation in the department	Open text	Not analyzed quantitatively

## Appendix B. STROBE Checklist for Cross-Sectional Studies

The checklist below (Table A2) records where each STROBE item for cross-sectional studies is addressed in the revised manuscript.

**Table A2:** Completed STROBE checklist for cross-sectional studies.

No.	STROBE Item	Recommendation	Reported in
1	Title and abstract	Study design indicated in title and abstract; balanced summary	Title; Abstract
2	Background/rationale	Scientific background and rationale explained	Section 1
3	Objectives	Specific objectives and pre-specified hypotheses stated	Section 1, final paragraphs
4	Study design	Key elements of design presented early	Section 2.1
5	Setting	Setting, locations and relevant dates given	Section 2.1 and Section 2.5
6	Participants	Eligibility criteria, sources and methods of selection	Section 2.1
7	Variables	Outcomes, exposures, predictors and effect modifiers defined	Section 2.2 and Section 2.3; Appendix A
8	Data sources/measurement	Sources of data and methods of assessment	Section 2.1 and Section 2.2
9	Bias	Efforts to address potential sources of bias described	Section 2.1 and Section 4.2
10	Study size	How the study size was arrived at	Section 2.1
11	Quantitative variables	Handling of quantitative variables and groupings explained	Section 2.1 and Section 2.2
12	Statistical methods	All methods, subgroups, missing data, sensitivity analyses	Section 2.4
13	Participants	Numbers at each stage; reasons for non-participation	Section 2.1
14	Descriptive data	Characteristics of participants; missing data	Section 3; Table 1
15	Outcome data	Numbers of outcome events or summary measures	Section 3; Table 2, Table 3, Table 4, Table 5, Table 6 and Table 7
16	Main results	Unadjusted and adjusted estimates with 95% confidence intervals	Section 3; Table 2, Table 4 and Table 6
17	Other analyses	Subgroup, interaction and sensitivity analyses	Section 2.4 and Section 3
18	Key results	Key results summarized with reference to objectives	Section 4.1 and Section 5
19	Limitations	Limitations, sources of bias and imprecision discussed	Section 4.2
20	Interpretation	Cautious overall interpretation	Section 4.1 and Section 5
21	Generalisability	External validity discussed	Section 4.2
22	Funding	Source of funding and role of funders	Funding statement
